# Two Homogametic Genotypes – One Crayfish: On the Consequences of Intersexuality

**DOI:** 10.1016/j.isci.2020.101652

**Published:** 2020-10-06

**Authors:** Tom Levy, Tomer Ventura, Giulio De Leo, Nufar Grinshpan, Faiza Amterat Abu Abayed, Rivka Manor, Amit Savaya, Menachem Y. Sklarz, Vered Chalifa-Caspi, Dan Mishmar, Amir Sagi

**Affiliations:** 1 Department of Life Sciences, Ben-Gurion University of the Negev, P.O. Box 653, Beer-Sheva 84105, Israel; 2 GenEcology Research Centre, School of Science and Engineering, University of the Sunshine Coast, Maroochydore, QLD 4558, Australia; 3 Hopkins Marine Station of Stanford University, Pacific Grove, CA 93950, USA; 4 Woods Institute for the Environment, Stanford University, Pacific Grove, CA 93950, USA; 5 The National Institute for Biotechnology in the Negev, Ben-Gurion University of the Negev, P.O. Box 653, Beer-Sheva 84105, Israel

**Keywords:** zoology, genetics, genotyping, evolutionary biology

## Abstract

In the Australian redclaw crayfish, *Cherax quadricarinatus* (WZ/ZZ system), intersexuals, although exhibiting both male and female gonopores, are functional males bearing a female genotype (WZ males). Therefore, the occurrence of the unusual homogametic WW females in nature is plausible. We developed W/Z genomic sex markers and used them to investigate the genotypic structure of experimental and native *C. quadricarinatus* populations in Australia. We discovered, for the first time, the natural occurrence of WW females in crustacean populations. By modeling population dynamics, we found that intersexuals contribute to the growth rate of crayfish populations in the short term. Given the vastly fragmented *C. quadricarinatus* habitat, which is characterized by drought-flood cycles, we speculate that intersexuals contribute to the fitness of this species since they lead to occasional increment in the population growth rate which potentially supports crayfish population restoration and establishment under extinction threats or colonization events.

## Highlights

• Two sexes and four sexual genotypes are consequences of crayfish intersexuality
• W/Z genomic sex markers were developed for the Australian redclaw crayfish
• Homogametic WW females were found for the first time in crayfish populations
• Intersexuals may contribute to fitness by increasing population growth rate

## Graphical Abstract

**Figure:**
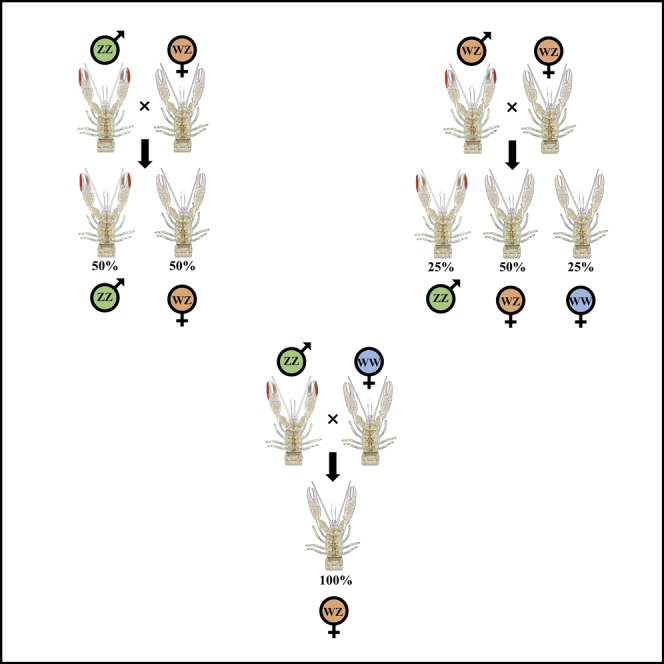

## Introduction

Intersexuality, a term first coined by Richard Goldschmidt (Goldschmidt, 1938; Reinboth, 1975), is not a deviated type of reproduction but is rather used to describe an individual of a gonochoristic species failing to fit into the typical sex definition of a male or a female. Instead, intersexuals may bear an unusual combination of male and female features such as sex chromosomes, genital opening, and gonads (Abdel-Moneim et al., 2015; Reinboth, 1975).

The occurrence of intersexuality in the animal kingdom is sometimes attributed to environmental effects or to external interventions, such as temperature (Olmstead and Leblanc, 2002; Olmstead and LeBlanc, 2007; Souissi et al., 2010), photoperiod (Dunn et al., 1993), pollutants (Barnhoorn et al., 2004; Ford et al., 2006), parasitism (Ford et al., 2007; Ianora et al., 1987), and bacterial infections (Rigaud and Juchault, 1998), and even to designated crossbreeding and hybridization of two species (Gomot, 1975). The natural occurrence of intersexuality is well established in some species—vertebrates and invertebrates alike (Adolfi et al., 2019; Armstrong and Marshall, 1964; Grilo and Rosa, 2017; Reinboth, 1975). In terms of fitness, previous studies have shown that intersexuality results in low fecundity, suggesting lower reproductive fitness for populations with higher occurrences of intersexuality (Ford et al., 2003, 2004; Jobling et al., 2002). It has also been suggested that morphological abnormalities in certain intersexuals impair mating success (Martins et al., 2009). However, although it is plausible that during evolution there is negative selection for intersexuals, the common occurrence of natural intersexuality could also suggest a possible fitness advantage.

For animals bearing the WZ/ZZ chromosomal system (WZ females and ZZ males), such as the fowl *Gallus gallus*, it has been suggested that the occurrence of the WW sex chromosome combination is lethal since most W-bearing primordial germ cells fail to differentiate into spermatozoa (Tagami et al., 1997). Similarly, in the fowl *Meleagris gallopavo*, WW blastoderms produced by parthenogenesis did not survive beyond two days of incubation (Harada and Buss, 1981). In contrast, laboratory experiments in crustaceans and amphibians involving artificial sex manipulations of a WZ female to a WZ male and crossing the WZ male with a normal WZ female resulted in a progeny that contains 25% of viable WW females. The sex manipulation was performed in isopod crustaceans using hormonal treatment (Juchault and Rigaud, 1995) and in the decapod *Macrobrachium rosenbergii* using implantation of the unique masculine crustacean androgenic gland (AG) or injection of AG cell suspension into females (Levy et al., 2016; Malecha et al., 1992), while testis implantation was used to masculinize females in frogs and salamanders (Humphrey, 1948; Mikamo and Witschi, 1964).

Natural occurrence of WW animals was suggested to exist in the branchiopod clam shrimp *Eulimnadia texana* (Weeks et al., 2010). However, this branchiopod is not gonochoristic but androdioecious hermaphrodite in which males and hermaphrodites coexist (Weeks et al., 2006). Additionally, it is noteworthy that unlike gonochoristic species bearing the W/Z system, the sex chromosomes in this androdioecious species are considered as sex chromosomes at a very early developmental stage (i.e. proto-sex chromosomes) (Weeks et al., 2010).

**Figure 1:**
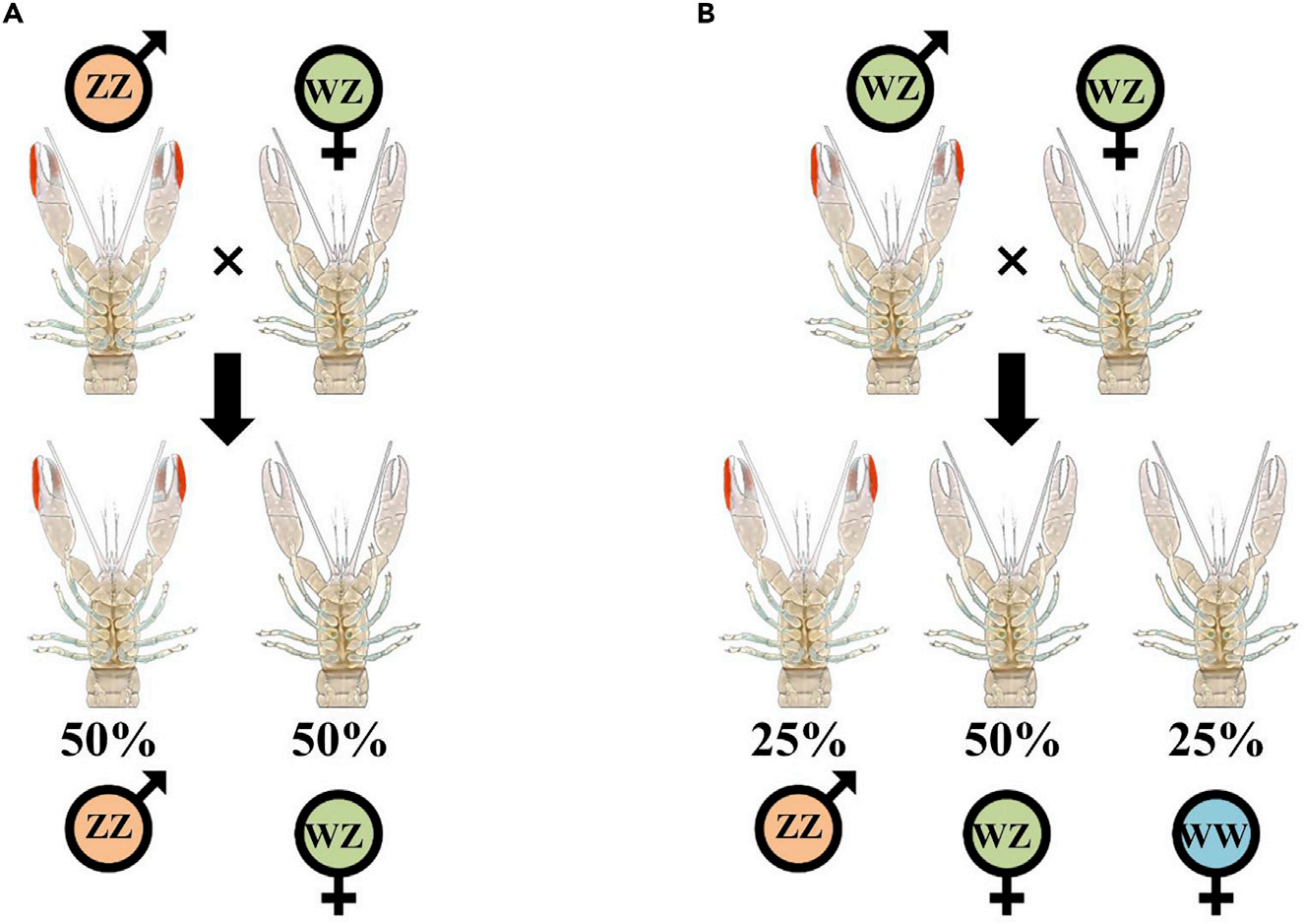
Crosses of (A) male and female and (B) intersex and female crayfish. The resulting genotype and phenotype ratios in each progeny are shown in the figure.

**Figure 2:**
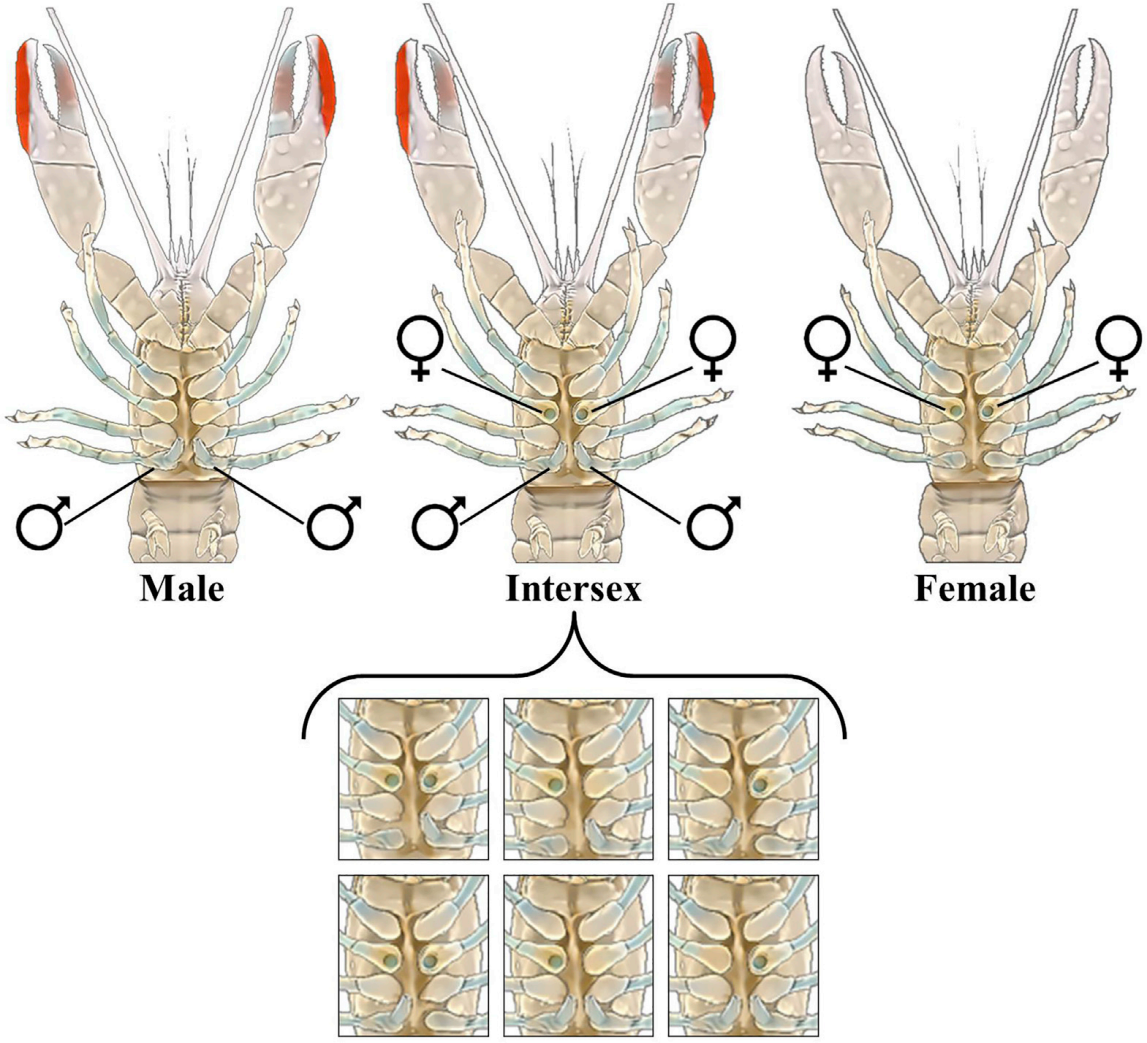
Genital Openings (Gonopores) in *C. quadricarinatus* Male – two gonopores at the base of the fifth pereiopod (left). Female – two gonopores at the base of the third pereiopod (right). Intersex – seven combinations of male and female gonopores observed and reported by Parnes et al., in 2003 (middle).

In the Australian redclaw crayfish, *Cherax quadricarinatus*, a gonochoristic species in which, according to progeny testing, sex is determined by the WZ/ZZ mode of inheritance (Parnes et al., 2003), a natural occurrence of 1–8% of intersexuals (i.e., bearing a combination of both male and female gonopores) within a given population has been reported (Brummett and Alon, 1994; Curtis and Jones, 1995; Sagi et al., 1996; Thorne and Fielder, 1991). In contrast to protandric hermaphrodite species in which male and female phases are well defined (except during episodic transitional phases between sexes [Benvenuto and Weeks, 2020]), intersexuals of the redclaw crayfish, although bearing both male and female gonopores, do not shift between sexes but are actually males with a female genotype (WZ males) (Parnes et al., 2003; Sagi et al., 1996). Anatomically, they have a constantly arrested ovary in a pre-vitellogenic state (Sagi et al., 1996, 2002). Morphologically, they exhibit male secondary characteristics, such as a red patch on the chela (Rosen et al., 2010; Sagi et al., 2002) and, like normal ZZ males, they present masculine behavior in terms of courtship and fighting (Barki et al., 2006). In a previous work that included progeny testing under laboratory conditions, it was shown that *C. quadricarinatus* intersexuals (WZ males) crossed with females (WZ) could skew the female:male ratio to 3:1 (Figure 1; [Parnes et al., 2003]). Also, when females from the progeny of such crosses were further crossed with normal males (ZZ), a third of them were viable WW females as indicated by their production of all-female progenies. In addition, seven types of *C. quadricarinatus* intersexuals, exhibiting seven different combinations of male and female gonopores, have been reported (Figure 2 and Sagi et al. 1996).

The above mentioned *C. quadricarinatus* WW females, similarly to other species in which WW females were achieved only through external intervention, have been reported only under laboratory conditions (Parnes et al., 2003). Although possible, even if existed in nature, according to the above reports, WW females in the laboratory are phenotypically indistinguishable from WZ females. To the best of our knowledge, the occurrence of WW females within gonochoristic species in nature has never been reported for any wild population in the animal kingdom. However, since intersexuals are found in wild populations of *C. quadricarinatus* (Curtis and Jones, 1995), the natural occurrence of Z-lacking WW females could be hypothesized. In such a case, questions could be asked regarding the essentiality of the Z chromosome in a WZ/ZZ sex determination system. Moreover, WW females, which should comprise third of the progeny of an intersexual crossed with a female (WZ × WZ), could cause a female bias in native populations.

In the current study, using genomic sex markers, we screened the genotypic composition of both cultured and native crayfish populations. This screening has enabled us to report for the first time Z-lacking WW females in native populations of *C. quadricarinatus*. We also modeled the population dynamics structure and tested the propensity of intersexuals and WW females to skew sex ratios in crayfish populations. Finally, in light of the potential contribution of intersexuals and WW females to the fitness of the species, we examined possible evolutionary advantages for female-biased populations, even though the notion is seemingly contradictory to the theory of sex allocation (Charnov, 1982).

## Results

### Sex-Specific Markers in *C. quadricarinatus*

**Figure 3:**
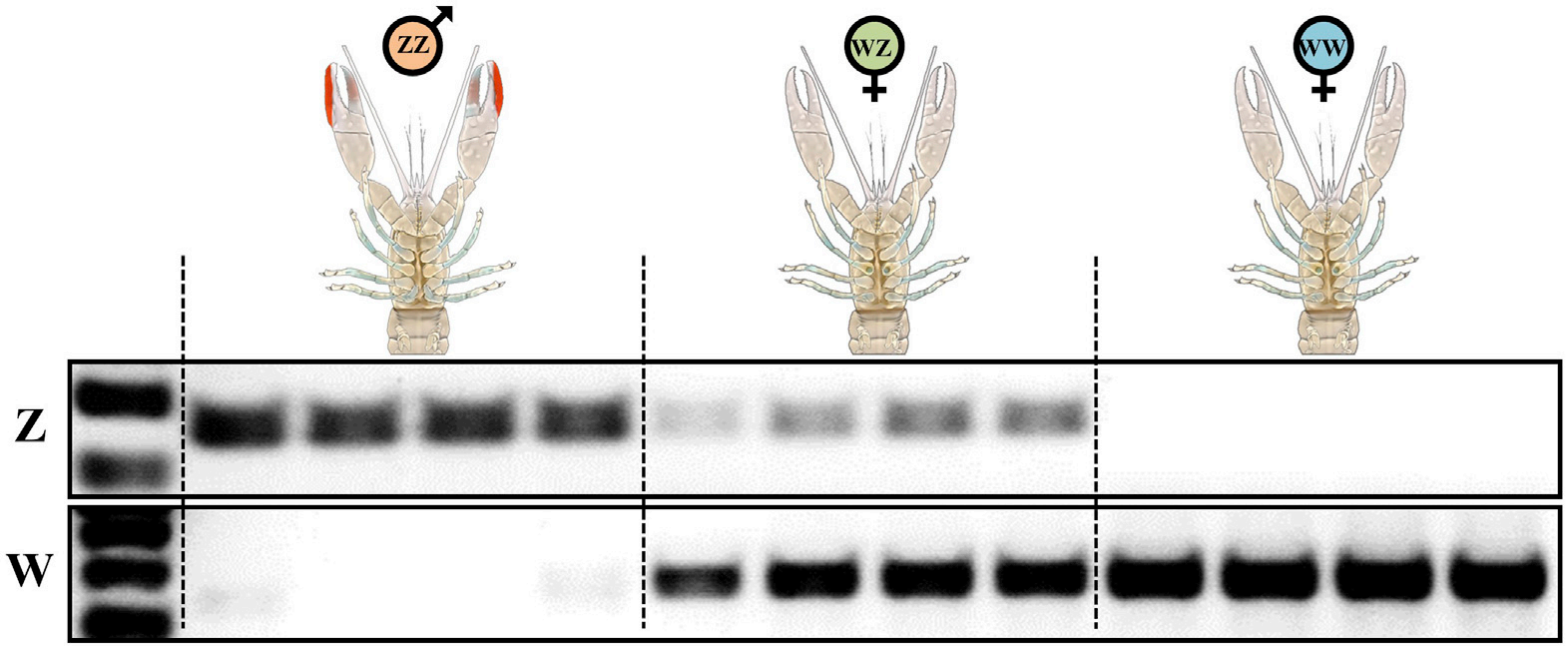
Identification of the Genotype of *C. quadricarinatus* Using Genomic Sex Markers DNA that was extracted from 4 animals of each genotype (ZZ, WZ, WW) was used as a template to amplify sex chromosome–specific markers (Z–top, W–bottom). A 100 bp DNA ladder is given in the left part of the gels.

Obtaining sex-specific markers in *C. quadricarinatus* was a crucial prerequisite for performing the different analyses in this study. To this end, restriction-site associated DNA sequencing (RADSeq; Davey and Blaxter, 2010) was applied to 12 male (ZZ) and 12 female (8 WZ + 4 WW) samples, resulting in a total of 50,052,191 single-end 90-bp sequence reads. Clustering of the reads yielded 147,952 unique RAD tags. To identify sex-specific tags, the reads of each sample were aligned to the RAD tags and quantified. RAD tags present in most (>60%) of the female samples but absent in all the male samples were considered as possible W-associated sex markers. Of the eight identified candidates (Table S1), 'Tag 906′ seemed most precise based on a preliminary polymerase chain reaction (PCR) screening. Therefore, this tag was extensively validated resulting with a W-specific marker, as attested by genotyping more than 1,500 animals in different populations coming from different continents (see details in the experiments below). Interestingly, the primers that amplified the W-band (120 bp) also amplified a Z-band (200 bp), as shown in Figure S1. Sequencing both W and Z products enabled us to design more specific primers, which amplified bands of higher intensity associated with either the W chromosome or the Z chromosome, separately, in thousands of animals that were different from those which were sampled for the RADSeq analysis (Figure 3).

### Genotyping the Various Intersexual Types

**Figure 4:**
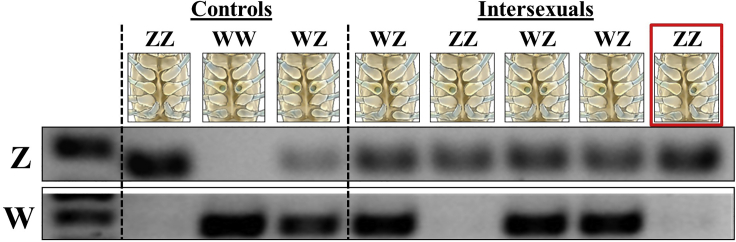
The Genotype of *C. quadricarinatus* Intersexuals Genomic sex markers were used to test the genotype of each intersex with every possible combination of female and male gonopores (right). The new type of intersexual that we found in this study, with one male and one female gonopore on the same side, is marked out with a red frame. ZZ male, WZ female, and WW female are given as controls. A 100 bp DNA ladder is given in the left part of the gels.

In addition to the intersexual types previously reported (Sagi et al., 1996), during the process of sampling the animals, we found a type of intersex individual bearing one male gonopore and one female gonopore on the same side of the animal. According to the sex-specific markers used in this study, intersexuals with 2 female and 1 or 2 male gonopores (n = 8) as well as intersexuals with 1 female gonopore and 1 male gonopore on opposite sides (n = 1) were found to bear a female WZ genotype. Intersexuals with 1 female and 2 male gonopores (n = 6) as well as intersexuals with 1 female gonopore and 1 male gonopore on the same side (n = 1) were found to bear the masculine ZZ genotype. Genotyping intersexuals bearing every possible male-female gonopore combination is shown in Figure 4.

### Progeny Testing of Females Crossed with Intersexuals

An examination of the progeny of females crossed with intersexuals (WZ × WZ) under laboratory conditions revealed that the observed ratios of WZ/ZZ:WW in the three progenies tested (3.8:1, 4.6:1, and 2.2:1) were not statistically different from the expected 3:1 ratio, according to the chi-square goodness of fit test (p *value* ≥ 0.16). Detailed results are presented in Table 1.

**Table 1:** Observed and Expected Genotypes in WZ × WZ (Intersex × Female) Crayfish Progenies

Progeny	Total N	Genotypic Composition Observed		Genotypic Composition Expected		p *value*
		WZ/ZZ	WW	WZ/ZZ	WW	
1	83	66 (80%)	17 (20%)	62 (75%)	21 (25%)	0.34
2	68	56 (82%)	12 (17%)	51 (75%)	17 (25%)	0.16
3	61	42 (69%)	19 (31%)	46 (75%)	15 (25%)	0.27

Values in the table are the number (percentage) of sampled animals from each progeny. p *values* represent the level of significance between the observed and expected results according to chi-square goodness of fit test.

### Intersex Progeny in Cultured Populations

Sex ratios were determined during the field experiments performed in earthen ponds in Australia and Israel. A clear 1:1 sex ratio was observed in control ponds stocked only with females and males, whereas ponds stocked only with females and intersexuals showed a clear female bias of a 5:1 ratio in Israel and ratios ranging from 2:1 to 3:1 in the Australian ponds. The sex-specific markers revealed that WW females were found in all treatment ponds, and the ratio of WZ to WW females was 4:1 in Israel and ranged from 2:1 to 4:1 in Australia (Table 2).

**Table 2:** Populations Cultured in Earthen Ponds in Israel and Australia

Pond	Stocked Phenotype			Phenotype of Sampled Progeny				Genotype of Sampled Females			
	Females	Males	Intersexuals	N	Females	Males	F:M Ratio	n	WZ	WW	WZ/WW Ratio
IL	20	0	5	52	43	9	5:1	43	35	8	4:1
AU 1	130	0	30	121	80	41	2:1	48	33	15	2:1
AU 2	130	0	30	144	109	35	3:1	48	32	16	2:1
AU 3	130	0	30	218	158	60	3:1	48	39	9	4:1
AU 4	130	30	0	160	80	80	1:1	20	20	0	–
AU 5	130	30	0	131	53	78	1:1	20	20	0	–

Four ponds were stocked with females and intersexuals (one as a preliminary experiment at Dor station, Israel [IL], and three at Cherax Park Aquaculture, Australia [AU 1–3]). Two control ponds were stocked with males and females at Cherax Park Aquaculture (AU 4–5). The number of sampled animals from each sex and the ratio between the two sexes are given.

### The Sexual Composition in Australian Crayfish Populations

**Figure 5:**
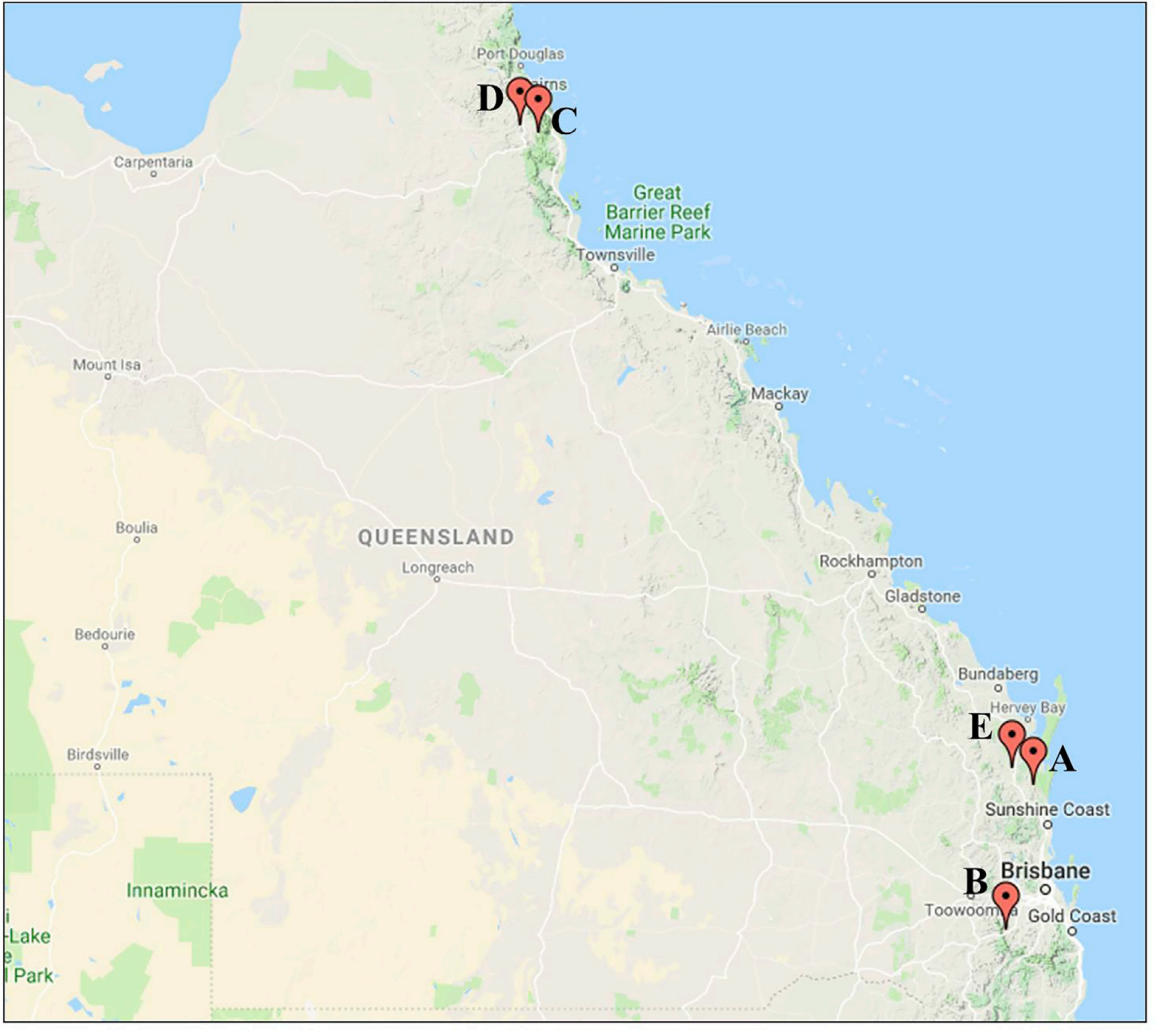
Sampling Locations in QLD, Australia (A–E) (A) Ironbark Redclaw Crayfish Farm, (B) Freshwater Australian Crayfish Traders Pty Ltd., (C) Klaus Cazzonelli Redclaw Farm, (D) AquaVerde Redclaw Hatchery & Farm, (E) Cherax Park Aquaculture. The map was created online through http://www.pinmaps.net.

An investigation of the sex ratio in native Australian crayfish populations at two different locations (A and B, shown in Figure 5) revealed that the female:male ratio was ∼1:1, with a very low proportion of intersexuals in the population (1.1 and 1.2%, respectively). Testing the sexual genotype of the females sampled at those two locations and at two more revealed that the samples from A, B, and C (but not from D) did indeed contain WW females, but only rarely, namely, 0.65, 1.05, and 0.7% of all the females sampled at locations A, B, and C, respectively. A detailed summary of the results for the native populations is presented in Table 3.

**Table 3:** Native Populations of the Australian Redclaw Crayfish

Location (See Figure 5)	Phenotype of Sampled Progeny				Genotype of Sampled Females			
	n	Female	Male	Male Intersexual	N	WZ	WW	WW [%]
A	852	419	423	10	307	305	2	0.65
B	570	287	276	7	287	284	3	1.05
C	-	-	-	-	286	284	2	0.70
D	-	-	-	-	294	294	0	0.00

Four locations in QLD, Australia, (see map, Figure 5) were examined. At two locations (A and B), animals were sampled at random and sorted phenotypically. Of the sampled animals, all or most of the females were genotypically tested. At the other two locations (C and D), the entire population was not examined, but only females were randomly sampled and genotypically tested. The number of females bearing each genotype and the percentage of WW females in the sampled population are given.

### Population Dynamics of *C. quadricarinatus*

A discrete-time and density-independent Malthusian growth model was developed to track the dynamics of the four phenotypes and genotypes in *C. quadricarinatus* populations, namely, ZZ males, WZ intersexuals (IS), WZ females, and WW females (for further details, see the Transparent Methods section in the supplemental information).

**Figure 6:**
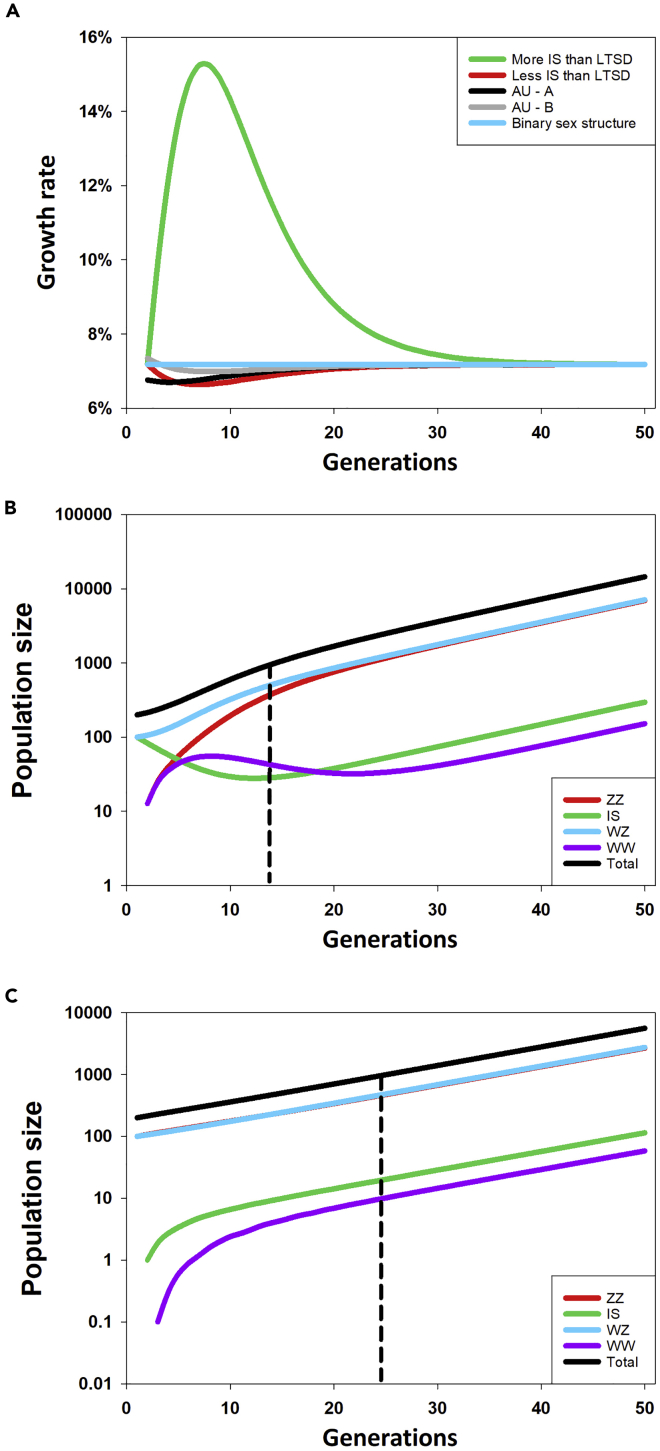
Simulated Dynamics of *C. quadricarinatus* Population Structure (A) Finite growth rate (rate of change in a generation) of crayfish populations as a function of time. Green line: a population with α = 4% (fraction of WZ females emerging as intersex IS) and the initial fraction of intersexuals in the founder population (i.e., at time t = 0) larger than that at the long-term stable distribution (LTSD) (IS_0_ = 50% > IS_∞_ and WZ_0_ = 50%, with ZZ_0_ = WW_0_ = 0); red line: same α as above but with the initial fraction of intersexuals in the founder population lower than that at LTSD (ZZ_0_ = 50% and WZ_0_ = 50%, with IS_0_ = WW_0_ = 0 < IS_∞_); black and gray lines: finite growth rate for two Australian native crayfish populations as described in Table 3 with an initial structure of N_0_ = [ZZ_0_ = 423, IS_0_ = 10, WZ_0_ = 417, WW_0_ = 2] and N_0_ = [ZZ_0_ = 276, IS_0_ = 7, WZ_0_ = 284, WW_0_ = 3] for AU-A and AU-B, respectively, and α = 4%; blue line: a population with a binary sex structure (no WZ progeny emerging as intersexuals, i.e., α = 0, only ZZ and WZ individuals). (B) Trajectories of population size in time for α = 4% and an initial fraction of intersexuals that is larger than that at the LTSD [IS_0_ = 100 (50%)>IS_∞_ and WZ_0_ = 100 (50%), with ZZ_0_ = WW_0_ = 0]. After an initial transitory period, depending upon the initial conditions, the population reaches a stable structure (i.e., a constant fraction of individuals in each class) while growing exponentially (long-term Malthusian growth in the semi-logarithmic diagram is represented by a straight line). (C) As in (B) but with an initial fraction of intersexuals that is lower than that at the LTSD [ZZ_0_ = 100 (50%) and WZ_0_ = 100 (50%), with IS_0_ = WW_0_ = 0 < IS_∞_]. The vertical dashed lines in B and C represent the generation in which the population size reaches or exceeds 1,000 individuals.

Simulations of *C. quadricarinatus* population dynamics (see Excel file: Data S1 in the supplemental information) showed that, regardless of the initial conditions (as long as the number of males [♂] = ZZ_0_+IS_0_ > 0 and the number of females [♀] = WZ_0_+WW_0_ > 0), the population reached a stable distribution (constant fraction of individuals for each phenotype/genotype) in about 30 generations (i.e., 15 years), with the fraction of individuals in the long run being ZZ_∞_ = 47.96%, WZ_∞_ = 48.96%, IS_∞_ = 2.04%, and WW_∞_ = 1.04%, respectively (Figure S2A). Simulations also showed that in the long term, the fraction of intersexual (IS) individuals will be about ½α, i.e., half the fraction of WZ progeny will emerge as intersexuals (α = 4%, while IS_∞_ = 2.04%), while the overall sex ratio (being the total number of functional males [ZZ + IS]: the total number of functional females [WZ + WW]) will be balanced at 1:1, as in a binary sex population, i.e., populations in which ZZ = 50%, WZ = 50%, and α = 0% (no WZ progeny emerging as intersexuals) (Figure S2B). The long-term annual growth rate of a population with a fraction α > 0 of WZ progeny emerging as intersexuals will be the same as that for a population with a binary sex structure (i.e., α = 0, only ZZ and WZ individuals, Figure 6A, blue line). In the short term, the growth rate of a population with α > 0 will be lower than the growth rate of a binary sex population (α = 0), if the initial fraction of intersexuals in the founder population (i.e., at time t = 0) is lower than that in the long-term stable distribution (LTSD), i.e., ZZ_0_ = 50% and WZ_0_ = 50%, with IS_0_ = WW_0_ = 0 < IS_∞_ and α > 0 (Figure 6A, red line). In contrast, in the short term, the annual growth rate for a population with α > 0 will be higher than that in the case of α = 0, if the fraction of intersexuals in the founder population (i.e., at time t = 0) is larger than that in the LTSD; for instance, for IS_0_ = 50% > IS_∞_ and WZ_0_ = 50%, with ZZ_0_ = WW_0_ = 0 (Figure 6A, green line). As a consequence, in the short term, the size of a population with α > 0 and initial conditions of intersexuals instead of ZZ males will grow much faster and the population will reach 1,000 individuals after as little as 15 years, in contrast to the 26 years required for the same population with ZZ males instead of intersexuals (Figures 6B and 6C). We note that the growth rate pattern of the two Australian native populations shown in Figure 6A (AU-A, black line and AU-B, gray line) is similar to the pattern of both the population with a binary sex structure (blue line) and the population with α > 0 and initial conditions of ZZ_0_ = 50% and WZ_0_ = 50%, with IS_0_ = WW_0_ = 0 < IS_∞_ (red line).

The actual fraction of individuals in each class in the short and long run depends on the specific values of the model parameters. However, the general properties listed above are invariant with respect to the actual values of the model parameters, i.e., finite growth rate (λ), survival of newborns (σ_0_), adult life expectancy (1/-ln(σ_a_)), per capita fecundity (ϕ), and the length of a generation (i.e., whether the time step represents ∼6 months, as assumed here). In particular, the LTSD, i.e., the relative size of each of the four classes, is uniquely a function of α and does not rely on the other model parameters. Further simulations showed that, in the long term, the population will tend to converge into a stable structure (i.e., fraction of individuals in each class) also under the assumption that the total number of individuals in the population remains constant (either because λ = 1 or because the population has reached the maximum carrying capacity and assuming that newborn individuals replace only the adults that die at each time step).

### Evolutionary Insight into Sexual Reproductive Strategies

**Figure 7:**
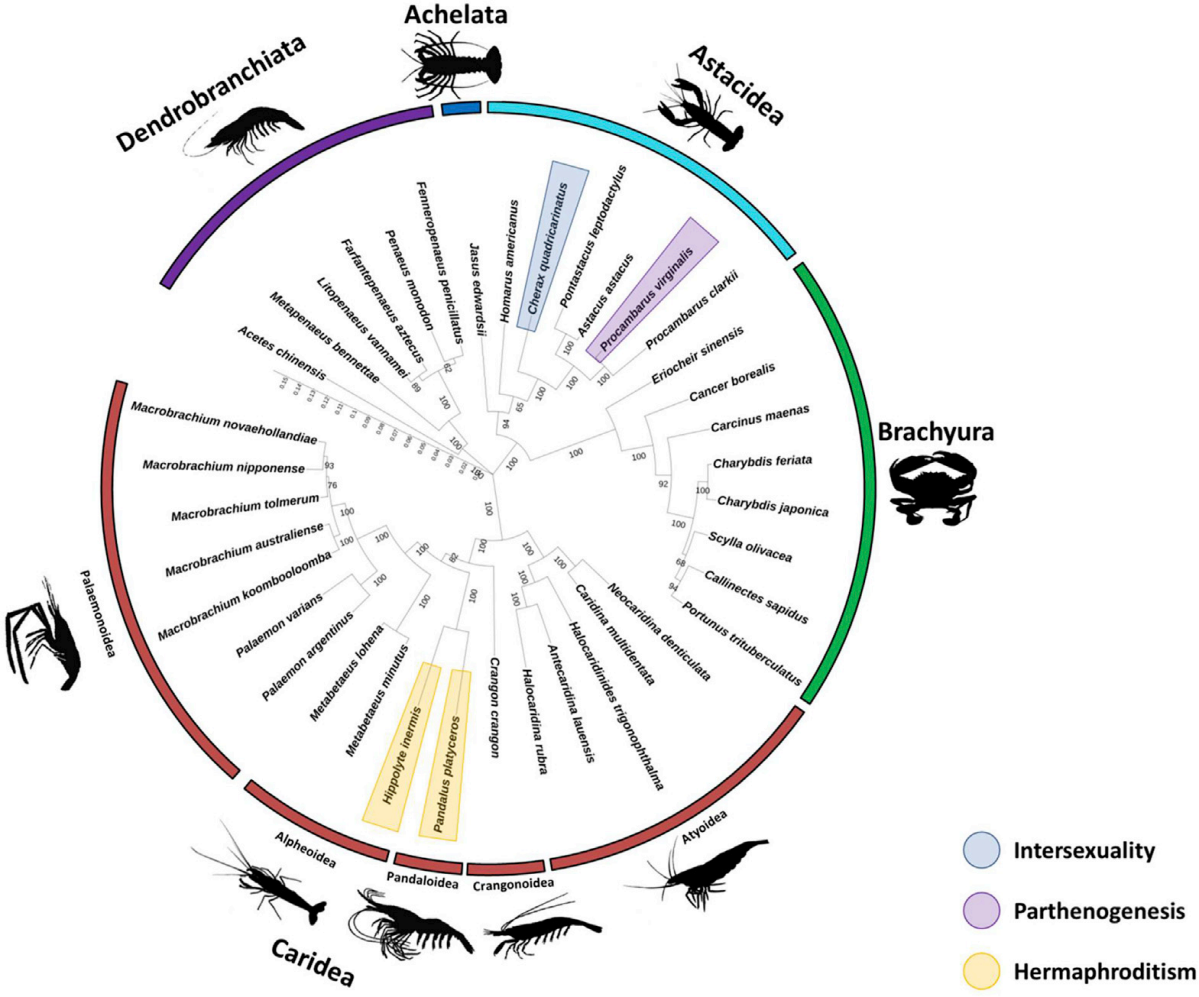
Phylogenetic Analysis of 38 Crustacean Species The evolutionary history was inferred by using the maximum likelihood method and Jones-Taylor-Thornton (JTT) matrix-based model (Jones et al., 1992). Supporting values (bootstrapping 1000 tests) and branch length scale are given. The infraorder of each species is indicated. Species with reproductive strategies other than gonochorism are colored.

According to our phylogenetic analysis (Figure 7), *C. quadricarinatus*, which exhibits a viable intersexual phenotype, is found within the Astacidea infraorder clade. To the best of our knowledge, among the species that have been analyzed, the only other astacidean species with a reproduction form that deviates from pure gonochorism (other than *C. quadricarinatus*) is the parthenogenetic marbled crayfish, *Procambarus virginalis*, in which a virginal form of reproduction occurs, resulting in an all-female clone (Martin et al., 2007). Out of the 38 decapod species used in our phylogenetic analysis, the two protandric hermaphrodite species, *Pandalus platyceros* (family Pandaloidea) and *Hippolyte inermis* (family Alpheoidea), were found to be phylogenetically closely related. However, both are within the Caridea infraorder clade, which is the furthest from the Astacidea clade, according to our phylogenetic tree. To summarize, species exhibiting hermaphroditism and the intersexual *C. quadricarinatus* are evolutionary distinct from each other.

## Discussion

To date, homogametic WW females in the WZ/ZZ sex determination system have been reported only following laboratory-induced manipulations (Levy et al., 2016; Malecha et al., 1992). The present study is the first to report the presence of naturally occurring WW females in native wild populations. The genomic sex markers established in this study – based on an experimental laboratory population of the redclaw crayfish in Israel – were found to be universal on the basis of validation in five geographically distinct native populations in Australia. Therefore, the markers facilitated, first, the screening of a large sample of animals from different locations, from both experimental and native populations, and, second, a comprehensive study eventually proved, for the first time, the existence of homogametic WW females in native *C. quadricarinatus* populations.

At first glance, the occurrence of WW females in native populations does not seem to be feasible because – extrapolating from avian species – the Z chromosome is believed to be vital in the WZ/ZZ sex determination scheme since WW animals were not developed (Harada and Buss, 1981; Tagami et al., 1997). However, previous studies of decapod crustaceans showed that artificial sex manipulations have yielded not only viable WW females (Levy et al., 2016, 2017; Malecha et al., 1992; Molcho et al., 2020) but also viable WW males lacking the masculine Z chromosome (Levy et al., 2019). Also, in the androdioecious hermaphrodite clam shrimp, WW hermaphrodites have male traits but lacking the Z proto-sex chromosome (Weeks et al., 2010). Therefore, in crustaceans, it is possible not only that the Z chromosome is not essential for life but also that it is not essential for masculine development. It is hence also possible that part of the sex determination and differentiation “tool kit” could instead reside in autosomal chromosomes. This notion has similarly been posited for the male-determining factors in some *Musca* flies (Sanchez, 2008) and for the autosomal *dpy-21* locus regulating the X chromosome in *Caenorhabditis elegans* (Meneely and Wood, 1984). In light of this this theory, it now becomes conceivable that in crustaceans viable WW females may occur in nature, as we did indeed find in *C. quadricarinatus* in the course of this study.

On the assumption that intersexuals and WW females do indeed exist in nature, one of the major questions raised in this study was whether they contribute to the fitness of the population. To address this question, we first confirmed that intersexuals were able to alter the common 1:1 female:male ratio in pond experiments in both Australia and Israel stocked with high fractions of intersexuals. The finding that this ratio ranged from 2:1 to 4:1 served as the first evidence that the presence of high fraction of intersexuals instead of males may alter the population sexual structure within a single generation. It is thus puzzling that while WW females produce all-female progenies and intersexuals produce female-biased progenies, native populations always showed a ∼1:1 female:male ratio, as described in the present study and by Bortolini et al. (2007). This conundrum could suggest that at low frequencies, intersexuals and WW females are not able to alter the sex ratio of *C. quadricarinatus* populations in the long term, as confirmed by our population dynamics model. Analysis of the population dynamics structure in terms of sex ratios of *C. quadricarinatus* populations with different initial conditions confirmed that irrespective of the initial ratios of ZZ males, WZ intersexuals, WZ females, and WW females, the long-term sex ratio was not biased, and the annual growth rate of the entire population converged to a constant value with a stable distribution of ZZ males, WZ females, WZ intersexuals, and WW females (approximately 48, 49, 2, and 1%, respectively). The prediction of our model, which showed that every *C. quadricarinatus* population, even if initiated with biased sex ratios, converged to a 1:1 female:male ratio, is in line with Fisher's principle claiming that this convergence is a result of natural selection because parental expenditure should be equal for both sexes (Fisher, 1930). In contrast, even if all crayfish populations converge in the long term to a 1:1 female:male ratio, our model suggests that occasionally an initial condition of high fractions of intersexuals contributes significantly to the annual increment of a given population in the short term. This result suggests that intersexuality and WW females might contribute to the fitness of this species under conditions of small founder populations, in which occurrences of intersexuals, and consequently of females, may result in a higher short-term population growth rate, contributing to colonization or restoration. Moreover, in a near extinction event, the higher the intersex ratio, the better the chances of the population to recover and reach steady state of equal sex ratios, according to Fisher's principle (Fisher, 1930).

All freshwater crayfish from Australia, New Zealand, New Guinea, and Madagascar belong to the same Parastacidae family, dating back hundreds of millions years to the Neoproterozoic era when Australia was part of the Gondwana supercontinent (McCormack, 2012). When Australia became dryer and warmer, crayfish populations experienced colonization events, became isolated, and evolved into the many crayfish species, including *C. quadricarinatus*, that are currently found across Australia (McCormack, 2012). In view of the vastly fragmented landscape with multiple drought-flood cycles where this species originated (Jones, 1990), it could be speculated that not only a fast growth rate and high fecundity (McCormack, 2012) but also intersexuality provided a competitive advantage for the settlement of *C. quadricarinatus* populations in new niches vs other crayfish species.

Although not related to intersexuality but rather to rearrangement of the sex chromosomes, some species of mammalian rodents (XX/XY system), including the African pygmy mouse (*Mus minutoides*) and several lemming species, exhibit frequent occurrence of females with a male genotype (XY females) (Bull and Bulmer, 1981; Fredga, 1983; Veyrunes et al., 2010). The contribution of mammalian XY females (the equivalent of intersexuals [WZ males] in *C. quadricarinatus*) to the fitness of the species is even more questionable than that of the WZ intersex crayfish since quarter of their progeny (YY genotyped) is lethal. However, since the progeny of such XY females is female biased, it was suggested that producing an excess of females could be an adaptive strategy for rapid recovery from low densities (Veyrunes et al., 2010). The latter is in line with our theory regarding the short-term advantage of female-biased crayfish populations.

Selection for a biased sex ratio could be also attributed to species whose sex is determined by environmental factors in which environmental conditions during embryogenesis affect the sex of the offspring. Under this assumption, an embryo will develop into a male phenotype under conditions where males have a higher fitness than females or vice versa under conditions in which females have a higher fitness than males (Uller et al., 2007). However, frequent fluctuations in the sex ratio in a given population, as a result of rapid changes in the environmental conditions, may also reduce the optimal matching of the offspring sex to the changed conditions (Uller et al., 2007) and, potentially, affect the fitness of the species. However, to the best of our knowledge, the occurrence of the intersexual phenomenon in *C. quadricarinatus* is not dependent on environmental factors.

In terms of morphology, in addition to the seven types of intersexuals reported before (Sagi et al., 1996), the present study revealed a type of intersex animal, which bears one female gonopore and one male gonopore on the same side. To date, only the genotype of the two most common intersexual types of *C. quadricarinatus*, those with two female and one or two male gonopores, proved to bear the WZ genotype, according to progeny testing (Parnes et al., 2003). However, genotype determination of each of the eight possible intersexual types is now possible as a result of genomic sex markers obtained in this study.

Unlike protandric hermaphrodite species, which episodically transform from one sex to the other (Bauer and Holt, 1998; Benvenuto and Weeks, 2020; Heath, 1977; Levy et al., 2020; Mutalipassi et al., 2018), the intersexual form in *C. quadricarinatus* uniquely represents a natural case of permanent co-occurrence of male and female characteristics within a gonochoristic scheme. The intersex animal is a functional male with a female genotype (WZ), bearing a combination of male and female gonopores and a mix of male and female reproductive systems (Khalaila et al., 1999; Parnes et al., 2003; Sagi et al., 1996). This intersexuality phenomenon has some similarities to gynandromorphism (i.e., bilateral manifestation of male and female phenotypes; [Farmer, 1972; Johnson and Otto, 1981; Micheli, 1991]) in other decapod crustaceans. However, gynandromorphs are the outcome of random and rare events of abnormalities produced through several possible mechanisms, including improper migration of chromosomes or cytogenetic complications in early embryonic development (Gjershaug et al., 2016), while *C. quadricarinatus* intersexuals are not rare and their fraction in the population is not random but relatively constant (Sagi et al., 1996; Webster et al., 2004). While intersexuality in *C. quadricarinatus* could be attributed to a simply random phenomenon, it could also potentially represent “evolution in the making” in which intersexuality is the current form in an evolutionary process from a gonochoristic mode of reproduction toward hermaphroditism or vice versa, as has been suggested to have occurred repeatedly in crustaceans along evolution (Benvenuto and Weeks, 2020). However, discussions of “evolution in the making” with respect to intersexuality are fraught with controversial theories regarding the direction of the transition process, suggesting either that the first crustacean lineage, the “ur-crustacean” (Hessler and Newman, 1975; Richter and Wirkner, 2020), had a gonochoristic mode of reproduction that changed to hermaphroditism at low population densities (Cisne, 1982) or vice versa (Hessler and Newman, 1975; Juchault, 1999).

The following line of thought should throw some light on the above argument: Reproductive strategies in the animal kingdom are highly diverse, ranging from gonochorism (Subramoniam, 2013) to different types of hermaphroditism (Bauer and Holt, 1998; Brook et al., 1994; de Almeida and Buckup, 2000; Levy et al., 2020) and even to parthenogenesis (Benvenuto and Weeks, 2020; Scholtz et al., 2003). Our phylogenetic analysis of available transcriptomes from different decapod species with respect to reproduction strategies indicates that *C. quadricarinatus* is found within the Astacidea clade, closely related to another *Cherax* species that also exhibits intersexual forms, *C. destructor* (Austin and Meewan, 1999) [*C. destructor* is missing from the phylogenetic analysis in the present study due to lack of transcriptional data in the National Center for Biotechnology Information (NCBI) server]. This clade is evolutionarily remote from the Caridea clade, which comprises a cluster of many decapod crustaceans exhibiting hermaphroditism (Baeza, 2007; Bauer and Holt, 1998; Benvenuto and Weeks, 2020; Subramoniam, 1981; Yaldwyn, 1966) but has a common ancestor with gonochoristic species. This finding is in agreement with a recent comprehensive phylogenomic analysis indicating that the Astacidea and Caridea infraorders diverged from a common ancestor ∼450 mya (Wolfe et al., 2019). Thus, if the intersexual phenomenon in *C. quadricarinatus* represents a case of “evolution in the making,” it most probably reflects a process of transition from gonochorism toward hermaphroditism rather than the opposite direction.

In summary, intersexuality in *C. quadricarinatus* represents a unique reproductive strategy, providing a window to peek into the evolutionary background of reproduction in crustaceans. To the best of our knowledge, this is the first gonochoristic species in the animal kingdom reported to contain both naturally occurring homogametic males and females (ZZ and WW, respectively). We speculate that the propensity of intersexuals and WW females to alter the sexual composition of *C. quadricarinatus* populations, and hence to increase the short-term population growth rate, might contribute to the fitness of this species and might assist it in colonization or in overcoming events of mass extinction. However, our findings do not resolve the open questions of whether this intersexual form contributes to fitness or just presents a random case of unusual reproduction.

### Limitations of the Study

Unlike animals within the X/Y mode of inheritance, in which the X chromosome is essential for life and YY animals are most likely inviable, and unlike avian species (W/Z system), in which the Z chromosome is vital, our study shows that in crustaceans bearing the W/Z mode of inheritance, viable WW females indeed exist. The latter raises questions regarding the genomic content of the sex chromosomes in crustaceans and calls for further investigation of sex determination within this W/Z system.

### Resource Availability

#### Lead Contact

Further information and requests should be directed to and will be fulfilled by the Lead Contact, Amir Sagi (sagia@bgu.ac.il).

#### Material Availability

This study did not generate new materials.

#### Data and Code Availability

All the data are available within the article.

## Methods

All methods can be found in the accompanying Transparent Methods supplemental file.

## Supplemental Information

Document S1. Transparent Methods, Figures S1 and S2, and Tables S1–S3

Data S1. The Excel File Used to Run the Population Dynamics Model, Related to Figure 6

## Footnotes

Supplemental Information can be found online at https://doi.org/10.1016/j.isci.2020.101652.
