# Supplementary material for: Two Homogametic Genotypes – One Crayfish: On the Consequences of Intersexuality
Source: iScience. 2020 Oct 6;23(11):101652. doi: 10.1016/j.isci.2020.101652 (PMC7578757; doi:10.1016/j.isci.2020.101652)
Supplement: Document S1. Transparent Methods, Figures S1 and S2, and Tables S1–S3 [file mmc1.pdf]

iScience, Volume *23*

Supplemental Information

Two Homogametic

Genotypes – One Crayfish:

On the Consequences of Intersexuality

Tom Levy, Tomer Ventura, Giulio De Leo, Nufar Grinshpan, Faiza Amterat Abu Abayed, Rivka Manor, Amit Savaya, Menachem Y. Sklarz, Vered Chalifa-Caspi, Dan Mishmar, and Amir Sagi

Table S1. W-associated candidate tags from RADSeq, Related to Figure 3.

Rad tag	M1	M2	M3	M4	M5	M6	M7	M8	M9	M10	M11	M12	F1	F2	F3	F4	F5	F6	F7	F8	F9	F10	F11	F12	Males	Females
	ZZ	ZZ	ZZ	ZZ	ZZ	ZZ	ZZ	ZZ	ZZ	ZZ	ZZ	ZZ	WZ	WZ	WZ	WZ	WZ	WZ	WZ	WZ	WW	WW	WW	WW		
Tag 906	0	0	0	0	0	0	0	0	0	0	0	0	18	106	0	39	45	81	136	13	1856	245	391	190	0	11
Tag 473	0	0	0	0	0	0	0	0	0	0	0	0	0	31	16	7	72	55	43	13	708	84	80	111	0	10
Tag 803	0	0	0	0	0	0	0	0	0	0	0	0	0	104	0	38	71	47	146	0	1045	235	115	105	0	9
Tag 935	0	0	0	0	0	0	0	0	0	0	0	0	7	67	0	13	24	47	114	0	1014	260	170	129	0	9
Tag 881	0	0	0	0	0	0	0	0	0	0	0	0	0	37	0	48	23	48	79	0	957	133	78	77	0	9
Tag 358	0	0	0	0	0	0	0	0	0	0	0	0	0	24	3	24	36	14	29	8	381	38	73	28	0	9
Tag 368	0	0	0	0	0	0	0	0	0	0	0	0	0	76	0	50	18	27	27	0	385	28	74	30	0	9
Tag 546	0	0	0	0	0	0	0	0	0	0	0	0	0	1	0	38	25	80	72	0	614	92	63	76	0	8
Total NGS reads	1,859,296	15,153,807	2,281,606	2,206,581	2,630,175	2,306,972	1,466,234	463,264	2,036,338	2,141,013	2,337,766	2,311,612	477,119	2,429,117	586,004	1,992,287	2,215,997	2,307,454	2,493,313	356,236	28,535,646	5,462,686	2,790,830	2,334,642		

NOTE.—The number of reads from each male (M) and female (F) that aligned to each RAD tag candidate is shown along with the total animals (males and females) in which the RAD tag appeared (i.e., >10 aligned reads). The total NGS reads that were sequenced per sample are given in the bottom row. The genotype of each sampled animal is denoted.

Table S2. Relationship between Mating Parents and their Progeny for the Australian Redclaw Crayfish, Related to Figure 6.

		Mating type			
		ZZ(♂)×WZ(♀)	IS(♂)×WZ(♀)	ZZ(♂)×WW(♀)	IS×WW(♀)
Mating frequency for ZZ and IS males	*p*	$\frac{ZZ}{ZZ + IS}$	$\frac{IS}{ZZ + IS}$	$\frac{ZZ}{ZZ + IS}$	$\frac{IS}{ZZ + IS}$
Reproductive output	*RO*	$\varphi \cdot P_{ZZ \times WZ} \cdot$ WZ	$\varphi \cdot P_{IS \times WZ} \cdot$ WZ	$\varphi \cdot P_{ZZ \times WW} \cdot$ WW	$\varphi \cdot P_{IS \times WW} \cdot$ WW
Progeny					
Males	ZZ	½ $RO_{ZZ \times WZ}$	¼ $RO_{IS \times WZ}$		
Intersexual	IS	α ½ $RO_{ZZ \times WZ}$	α ½ $RO_{IS \times WZ}$	α $RO_{ZZ \times WW}$	α ½ $RO_{IS \times WW}$
Females	WZ	(1-α) ½ $RO_{ZZ \times WZ}$	(1-α) ½ $RO_{IS \times WZ}$	(1-α) $RO_{ZZ \times WW}$	(1-α) ½ $RO_{IS \times WW}$
WW females	WW		¼ $RO_{IS \times WZ}$		½ $RO_{IS \times WW}$

NOTE.—φ is the per capita fecundity (egg per WZ and WW female per reproductive event) and α the fraction of WZ progeny emerging as an intersexual (IS).

Table S3. List of the Genes used for the Phylogenetic Analysis, Related to Figure 7.

#	Gene
1	Serine tRNA ligase
2	Splicing factor 3B subunit 2
3	40S ribosomal protein S3a
4	60S ribosomal protein L8
5	4-hydroxybenzoate polyprenyltransferase, mitochondrial
6	Stomatin-like protein 2, mitochondrial
7	V-type proton ATPase subunit H
8	40S ribosomal protein S24
9	Coatomer subunit beta
10	Dihydrolipoyl dehydrogenase, mitochondrial
11	2-oxoglutarate dehydrogenase complex component E2

NOTE.—Out of 87 BUSCOs shared by all 38 crustacean transcriptomes, the 11 proteins indicated above appeared only once per transcriptome. These proteins were further concatenated and used for phylogenetic analysis.

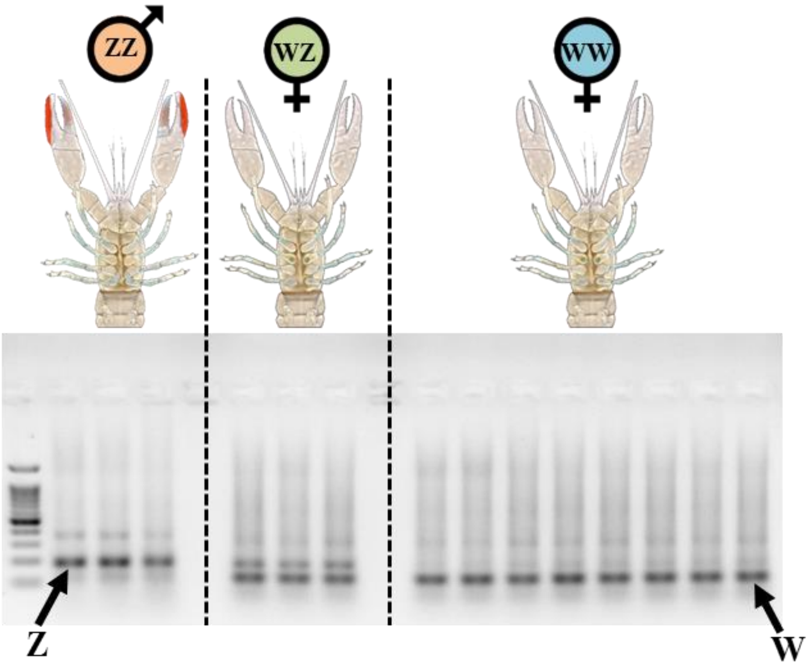

Figure S1. Tag 906 in *C. Quadricarinatus*, Related to Figure 3. DNA that was extracted from 3 ZZ males, 3 WZ females and 8 WW females was used as a template to amplify Tag 906 which resulted in a lower 120 bp W-band and a higher 200 bp Z-band. A 100 bp DNA ladder is given in the left part of the gel.

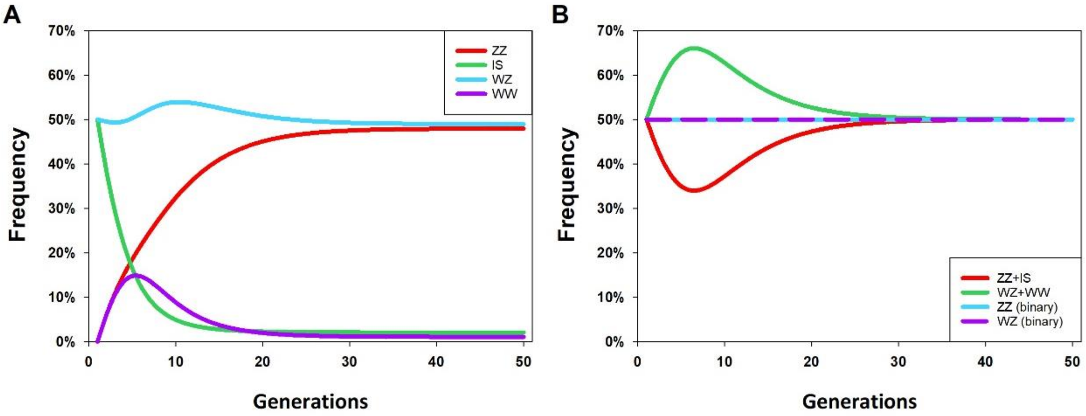

Figure S2. Simulated dynamics of *C. quadricarinatus* population structure, Related to Figure 6. (A) Population structure [ZZ males (red), IS male-intersexuals (green), WZ females (blue) and WW females (purple)] as a function of time for α = 4% (fraction of WZ females emerging as IS) with initial fraction of male-intersexuals in the founder population (i.e., at time t = 0) larger than the long term stable distribution (LTSD) (IS_0 = 50% > IS_∞ and WZ_0 = 50%, with $ZZ_0 = WW_0 = 0$). (B) Fraction of ZZ + IS males (red) and WZ + WW females (green) in a population with α = 4% with initial fraction of male-intersexuals larger than the LTSD (IS_0 = 50% > IS_∞ and WZ_0 = 50%, with ZZ_0 = WW_0 = 0). In a binary population (i.e., α = 0, ZZ and WZ individuals only), the sex ratio is 1:1 (blue and dashed purple lines).

Transparent Methods

Animals

Cherax quadricarinatus animals (used for DNA extraction, analysis of sex-specific markers and progeny testing) were reared in 600-L tanks at 26±2 °C with constant aeration, a light regime of 14:10 (L:D) and food (shrimp pellets comprising 30% protein) *ad libitum* at Ben-Gurion University of the Negev (BGU), Israel.

Identification of genomic sex markers in *C. quadricarinatus*

C. quadricarinatus DNA was extracted from 12 males (ZZ) and 12 females (8 WZ and 4 WW, confirmed by progeny testing). Briefly, muscle tissue was dissected from each animal and frozen using liquid nitrogen. Then, the tissue was ground with a mortar and pestle, and the DNA was extracted with a DNeasy Blood & Tissue DNA isolation kit (Qiagen, Venlo, Netherlands), according to the manufacturer's instructions. The DNA samples were sent to Floragenex (Portland, OR, USA) for restriction site-associated DNA sequencing (RADSeq) using Illumina technology. Subsequent bioinformatics analysis was carried out at the Bioinformatics Core Facility of BGU using NeatSeq-Flow (Sklarz et al., 2018) and additional R scripts. Since the quality of the reads, determined with FastQC version 0.11.2, was satisfactory, no further trimming was performed. Reads from female samples were clustered at 98% similarity using vsearch (Rognes et al., 2016). All reads were aligned against the resulting RAD tags with bowtie2 using the "--very-sensitive preset" option. Reads with mapping quality below 10 were discarded. The number of reads per sample that mapped to each RAD tag was obtained with the idxstats tool included in samtools 1.3 (Li et al., 2009). A tag was considered as a W-associated candidate if it had more than 10 mapped reads in 60% or more (i.e., > 7 animals) of the female samples but did not appear in any of the male samples.

To verify the sex-specific markers identified by RADSeq, 4 μL of gDNA of male and female *C. quadricarinatus* animals (differed from those which were sampled for the RADSeq

analysis) as a template were amplified by PCR (94 °C for 3 min, followed by 35 cycles of 94 °C for 30 s, 55 °C for 30 s, and 72 °C for 45 s, and then a final elongation step of 72 °C for 10 min) with 1 µL of forward primer, 1 µL of reverse primer (10 µM each), 12.5 µL of Ready Mix REDTaq (Sigma) and water to a final volume of 25 µL. PCR products were separated on 2% agarose gel stained with SYBR Safe DNA gel stain (Invitrogen, Carlsbad, CA, USA). Bands were excised from the gel, isolated using NucleoSpin Gel and PCR Clean-up kit (Macherey-Nagel, Düren, Germany), and sent for Sanger sequencing. The tag validated as a sex-specific marker was used to test the genotypes of the animals in the different experiments described in this study and also to reveal the genotypes of the different types of intersexuals.

Progeny testing

Adult intersexuals with two female gonopores and one male gonopore (the mostly common gonopore combination that was available in our experimental population) were bred with females in a communal 600-L tank. Upon fertilization, three berried females were transferred to individual 100-L tanks. After hatching, a sample of animals from each progeny ($n_1 = 83$, $n_2 = 68$, $n_3 = 61$) was taken, and the genotype of each sampled animal was determined using the sex-specific markers described above. The observed ratio of WZ/ZZ to WW animals was compared to the expected ratio (3:1) using the chi-square test for goodness of fit.

Field experiments in earthen ponds

As a preliminary experiment, 20 females and 5 intersexuals (each bearing two female gonopores and one male gonopore) were stocked in a 350-m^2 earthen pond at the Aquaculture Research Station, Dor, Israel. All animals weighed between 60 to 100 g. During the five months of grow-out, from May to November, the animals were fed *ad libitum* and water temperature was maintained at 25 ± 4 ºC. In addition, a comparative study of females stocked with males vs females stocked with intersexuals was conducted at the Cherax Park Aquaculture farm, Queensland, Australia. In this experiment, three 375-m^2 earthen ponds were stocked with 130

females and 30 intersexuals (treatment), while two ponds were stocked with 130 females and 30 males (control). Females were in the weight range of 65 to 100 g, and males/intersexuals, 120 to 150 g. During the 6 months of grow-out, from July to December, the animals were fed *ad libitum* and water temperature was maintained at 25 ± 4 ºC. At the end of the grow-out period, both in Israel and Australia, the animals harvested, and the progeny (animal weight ranging from 9 to 60 g) were phenotypically sex sorted by assessment of male and female gonopores. To test whether the females were WZ or WW, the sex markers described above were used. In Israel, all females (n = 43) were genetically tested, while in Australia, 48 females from each treatment pond and 20 females from each control pond were tested.

WW females in native Australian crayfish populations

During this study, we did not have access to or permission to sample wild populations in Australian rivers. Therefore, to reliably assess the native crayfish population, we sampled populations of natively farmed crayfish that had been introduced from the wild eight years prior to sampling and believed not to have artifacts of inbred lines. To determine whether there were WW females in those populations, animals were sampled from four locations. In two locations (A and B; Figure 3) we had access to the entire population. For those populations, 852 animals from A and 570 from B were sorted for sex, and of those 307 and 287 females, respectively, were genetically tested using our sex markers. In the other two locations (C and D; Figure 3), males and females were separated in advance, so we could not examine the sex ratio of the entire population, but we did sample 286 and 294 females to test their sexual genotype (WW or WZ).

Modelling population dynamics of *C. quadricarinatus*

We developed a simple demographic model to simulate discrete-time, density-independent Malthusian growth of *C. quadricarinatus* populations and to track the dynamics of the four

phenotypes-genotypes, namely, ZZ males, WZ intersexuals (designated IS), and WZ and WW females.

We assumed that the mean generation time, i.e., the mean age at which females give birth, to be equal to 6 months, and the length of the time step *t* was set accordingly. We also assumed that the fraction σ_o of the progeny generated at time *t* and surviving to the next time step would be reproductive 6 months later, namely, at time *t+1*. The species-specific maximum life span of *C. quadricarinatus* depends on environmental (temperature, salinity, pH, water quality, etc.) and ecological (productivity, presence of predators, etc.) conditions. Field studies have provided evidence of *C. quadricarinatus* populations with a life span of about 5 years (Jones, 1990). To account for the higher mortality of younger/smaller individuals, we assumed that the mean life expectancy *LE*, i.e., the mean number of years that a 1-year old crayfish could be expected to live, was about half the life span, i.e., 2.5 years; we then computed the natural mortality rate μ [year^{-1}] as 1/*LE* and derived the 6-month survival, i.e., the fraction σ_A of adult crayfish surviving between time *t* and time *t+1*, as $\sigma_A = \sqrt{e^{-\mu}} = 81.9\%$. We assumed a rapidly growing population capable of doubling its abundance in a 10-generation time span, i.e., about 5 years, which corresponds to a 6-month finite growth rate (λ) of $\lambda = 2^{\frac{1}{10}} = 1.0718$, i.e., a 7.18% of population increment in 6 months or, equivalently, a 14.88% increment per year. We assumed a per-capita fecundity of φ=300 eggs per reproductive event for both WZ and WW females [which is within the range of previous reports (Curtis and Jones, 1995)], and set the fraction of eggs hatching and surviving to reproductive maturity 6 months later as σ_o = (λ - σ_A)/(½ φ) = 0.169%, which is consistent with a 7.18% population growth rate in 6 months. We assumed that at each time step adult WZ and WW females in the population are fertilized by males and intersexuals in proportion to the males' relative abundance in the population, namely ZZ/(ZZ+ IS) and IS/(ZZ+ IS), respectively. A small fraction α of the WZ progeny will emerge as intersexuals (IS). While according to the literature IS proportion can range from 1% to 8%; (Brummett and Alon, 1994; Curtis and Jones, 1995; Sagi et al., 1996; Thorne and Fielder, 1991), most of the published data report on 4% of WZ progeny that emerges as intersexuals.

Therefore, in most scenarios described below we have used α=4%. However, since it is unknown whether this value is fixed within given populations and the genetic basis behind the intersexuality phenomenon is not clear, we allowed the option of changing the α in the model's Excel file provided with this article (Data S1). The formulas for deriving the reproductive output in each mating type, given the abundance of males and females in the brood stock, are given in Table S2. Accordingly, the equation describing the dynamics of the population may be expressed as follows:

$$\mathbf{N}_{t+1} = \mathbf{M} \times \mathbf{N}_t + \sigma_o \cdot \mathbf{R}_t \quad (1)$$

where:

- $\mathbf{N}_t = [ZZ_t, IS_t, WZ_t, WW_t]$ is the vector with the number of individuals of type ZZ, IS, WZ and WW, respectively, at time *t*, just before reproduction;
- **M** is a 4 × 4 square matrix with sub-diagonal elements $m_{ij} = \sigma_A = 0.819$ (i = 2..4, j = 1..3), zero otherwise, “×” indicates matrix multiplication;
- σ_o = 0.169% is the fraction of eggs that hatch and survive to the next generation;
- $\mathbf{R}_t$ is the vector for egg reproductive output at time *t* for each type computed by using the reproduction formula reported in Table S2, namely:

$$R_t^{ZZ} = \frac{1}{2}\varphi\frac{ZZ_t}{ZZ_t + IS_t}WZ_t + \frac{1}{4}\varphi\frac{IS_t}{ZZ_t + IS_t}WZ_t = \frac{1}{2}\varphi WZ_t\left(\frac{ZZ_t}{ZZ_t + u \cdot IS_t} + \frac{1}{2}\frac{IS_t}{ZZ_t + IS_t}\right) \quad (2a)$$

$$R_t^{IS} = \alpha\frac{1}{2}\varphi\frac{ZZ_t}{ZZ_t + IS_t}WZ_t + \alpha\frac{1}{2}f\frac{IS_t}{ZZ_t + IS_t}WZ_t + \alpha\varphi\frac{ZZ_t}{ZZ_t + IS_t}WW_t + \alpha\frac{1}{2}\varphi\frac{IS_t}{ZZ_t + IS_t}WW_t$$

$$= \alpha\frac{1}{2}\varphi\left(WZ_t + \frac{2 \cdot ZZ_t + IS_t}{ZZ_t + IS_t}WW_t\right) \quad (2b)$$

$$R_t^{WZ} = (1 - \alpha)\frac{1}{2}\varphi\left(WZ_t + \frac{2 \cdot ZZ_t + IS_t}{ZZ_t + IS_t}WW_t\right) \quad (2c)$$

$$R_t^{WW} = \frac{1}{4}f\frac{IS_t}{ZZ_t + IS_t}WZ_t + \frac{1}{2}f\frac{IS_t}{ZZ_t + IS_t}WW_t = \frac{1}{2}f\frac{IS_t}{ZZ_t + IS_t}\left(\frac{1}{2}WZ_t + WW_t\right) \quad (2d)$$

where φ = 300 eggs is the per-capita female fecundity, ½ accounts for the sex ratio, and α = 4%.

Population dynamics were simulated for 50 generations (25 years) with α, the fraction of WZ animals emerging as IS, set either to 4% or 0% (the 0% is representing a population generated by only males and females but no intersexuals). For initial conditions (i.e., number of individuals in each class at time t = 0), we considered four cases, two hypothetical cases, with the fraction of intersexuals in the population being either larger or smaller than the fraction of intersexuals at the long term stable distribution (LTSD), and two cases representing the estimated population structure for two of the native Australian populations sampled in this study (populations A and B described in Table 3), as follows:

- 0% intersexuals (all males are ZZ); $\mathbf{N}_0 = [ZZ_0 = 100, IS_0 = 0, WZ_0 = 100, WW_0 = 0]$.
- 50% intersexuals (all males are WZ); $\mathbf{N}_0 = [ZZ_0 = 0, IS_0 = 100, WZ_0 = 100, WW_0 = 0]$.
- AU – A ; $\mathbf{N}_0 = [ZZ_0 = 423, IS_0 = 10, WZ_0 = 417, WW_0 = 2]$.
- AU – B ; $\mathbf{N}_0 = [ZZ_0 = 276, IS_0 = 7, WZ_0 = 284, WW_0 = 3]$.

For the four scenarios above, the fraction α of WZ animals emerging as IS was set to 4%. We also ran an additional hypothetical simulation for $\alpha = 0\%$ (intersexuals cannot occur) to represent the case of 1:1 males:females sex ratio. The Excel file used to run the model is available in the online supplementary information (Data S1).

Phylogenetic analysis of decapod crustaceans

The identification of orthologs from diverse crustaceans and their multiple sequence alignment were carried out at the Bioinformatics Core Facility of BGU using the NeatSeq-Flow platform (Sklarz et al., 2018). The transcriptomes of all decapod crustacean species that were available in NCBI (http://www.ncbi.nlm.nih.gov) were downloaded (36 decapod species) ("NCBI,") ("NCBI,"). Two more transcriptomes, one from *Pandalus platyceros* and the other from *Hippolyte inermis*, had been assembled in currently performed studies in our laboratory. All 38 transcriptomes were translated with Transdecoder version 5.5.0 (Haas and Papanicolaou, 2016), and the protein sequences of the longest open reading frames (ORFs) for each transcript were used for further analysis. BUSCO analysis (Simão et al., 2015) was performed on all

transcriptomes, using “--mode transcriptome” against the Metazoa database (metazoa_odb9). An R script was used to select BUSCO accessions that appeared at least once in all transcriptomes, resulting in 87 (of 978) “shared BUSCOs.” The protein sequences of the 87 shared BUSCOs were extracted from the “ancestral” file of the metazoa_odb9 database. A blast database was constructed for each of the transcriptome predicted protein sets, and blastp version 2.7.1+ (Altschul et al., 1997) was used to find the predicted proteins matching the shared BUSCO protein sequences. The blastp result tables were processed with parse_blast (https://github.com/bioinfo-core-BGU/parse_blast) with the following arguments: --max_evalue 1e-10 --min_align_len 60 --min_coverage 70 --min_pident 50. For each shared BUSCO, matched protein sequences from all transcriptomes were extracted using samtools faidx version 1.3 (Li et al., 2009). Out of the 87 protein sequences, 11 (Table S3) appeared only once in each of the 38 transcriptomes. We have used only proteins that were represented once to avoid the complications of analyzing the presence of isoforms. The sequences of these 11 proteins were concatenated and submitted to multiple sequence alignment with MAFFT version 7.427 (Katoh and Standley, 2013). To find the best phylogenetic model to select with likelihood-based criteria, we used the Smart Model Selection (SMS) function with Bayesian information criterion (BIC) in PhyML (Lefort et al., 2017). Finally, the evolutionary history was inferred by the maximum likelihood method and the Jones-Taylor-Thornton (JTT) matrix-based model (Jones et al., 1992) with bootstrapping 1000 replicates. The evolutionary phylogenetic analysis was conducted and visualized using MEGA X (Kumar et al., 2018).

Supplemental References

NCBI [Online]. Available: http://www.ncbi.nlm.nih.gov/.

Altschul, S. F., Madden, T. L., Schaffer, A. A., Zhang, J. H., Zhang, Z., Miller, W. and Lipman, D. J. (1997). Gapped BLAST and PSI-BLAST: a new generation of protein database search programs. Nucleic Acids Res. 25**,** 3389-3402.

Brummett, R. E. and Alon, N. C. (1994). Polyculture of nile tilapia (*Oreochromis niloticus*) and australian red claw crayfish (*Cherax quadricarinatus*) in earthen ponds. Aquaculture 122**,** 47-54.

Curtis, M. C. and Jones, C. M. (1995). Observations on monosex culture of redclaw crayfish *Cherax quadricarinatus* von Martens (Decapoda: Parastacidae) in earthen ponds. J. World Aquacult. Soc. 26**,** 154-159.

Haas, B. and Papanicolaou, A. 2016. *TransDecoder (find coding regions within transcripts)* [Online]. Available: http://transdecoder.github.io.

Jones, C. M. (1990). The biology and aquaculture potential of the tropical freshwater crayfish, *Cherax quadricarinatus* (Department of Primary Industries Queensland).

Jones, D. T., Taylor, W. R. and Thornton, J. M. (1992). The rapid generation of mutation data matrices from protein sequences. Comput. Appl. Biosci. 8**,** 275-282.

Katoh, K. and Standley, D. M. (2013). MAFFT multiple sequence alignment software version 7: Improvements in performance and usability. Mol. Biol. Evol. 30**,** 772-780.

Kumar, S., Stecher, G., Li, M., Knyaz, C. and Tamura, K. (2018). MEGA X: molecular evolutionary genetics analysis across computing platforms. Mol. Biol. Evol. 35**,** 1547-1549.

Lefort, V., Longueville, J. E. and Gascuel, O. (2017). SMS: Smart model selection in PhyML. Mol. Biol. Evol. 34**,** 2422-2424.

Li, H., Handsaker, B., Wysoker, A., Fennell, T., Ruan, J., Homer, N., Marth, G., Abecasis, G., Durbin, R. and Proc, G. P. D. (2009). The sequence alignment/map format and SAMtools. Bioinformatics 25**,** 2078-2079.

Rognes, T., Flouri, T., Nichols, B., Quince, C. and Mahe, F. (2016). VSEARCH: a versatile open source tool for metagenomics. Peerj 4.

Sagi, A., Khalaila, I., Barki, A., Hulata, G. and Karplus, I. (1996). Intersex red claw crayfish, *Cherax quadricarinatus* (von Martens): Functional males with pre-vitellogenic ovaries. Biol. Bull. 190**,** 16-23.

Simão, F. A., Waterhouse, R. M., Ioannidis, P., Kriventseva, E. V. and Zdobnov, E. M. (2015). BUSCO: assessing genome assembly and annotation completeness with single-copy orthologs. Bioinformatics 31**,** 3210-3212.

Sklarz, M. Y., Levin, L., Gordon, M. and Chalifa-Caspi, V. (2018). Neatseq-flow: A lightweight high throughput sequencing workflow platform for non-programmers and programmers alike. bioRxiv**,** 173005.

Thorne, M. J. and Fielder, D. R. (1991). The red cuticle on the claw of male Cherax quadricarinatus (Decapoda: Parastacidae). Mem. Queensl. Mus. 31**,** 277.
